# Identification of Functional CircRNA–miRNA–mRNA Regulatory Network in Dorsolateral Prefrontal Cortex Neurons of Patients With Cocaine Use Disorder

**DOI:** 10.3389/fnmol.2022.839233

**Published:** 2022-04-14

**Authors:** Yun Chen, Xianfeng Li, Shiqiu Meng, Shihao Huang, Suhua Chang, Jie Shi

**Affiliations:** ^1^Department of Pharmacology, School of Basic Medical Sciences, Peking University Health Science Center, Beijing, China; ^2^Beijing Key Laboratory on Drug Dependence Research, National Institute on Drug Dependence, Peking University, Beijing, China; ^3^Department of Gastroenterology of Dapping Hospital, Third Military Medical University, Chongqing, China; ^4^Key Laboratory of Molecular Epidemiology of Hunan Province, School of Medicine, Hunan Normal University, Changsha, China; ^5^Institute of Mental Health, National Clinical Research Center for Mental Disorders, Key Laboratory of Mental Health and Peking University Sixth Hospital, Peking University, Beijing, China; ^6^Peking University, Shenzhen Hospital, Shenzhen, China

**Keywords:** cocaine use disorder, circRNAs, ceRNAs regulatory network, protein–protein interaction network, hub genes, integrated bioinformatics analysis

## Abstract

Increasing evidence has indicated that circular RNAs (circRNAs) act as competing endogenous RNAs (ceRNAs) regulatory network to regulate the expression of target genes by sponging microRNAs (miRNAs), and therefore play an essential role in many neuropsychiatric disorders, including cocaine use disorder. However, the functional roles and regulatory mechanisms of circRNAs as ceRNAs in dorsolateral prefrontal cortex (dlPFC) of patients with cocaine use disorder remain to be determined. In this study, an expression profiling for dlPFC in 19 patients with cocaine use disorder and 17 controls from Gene Expression Omnibus datasets was used for the differentially expressed circRNAs analysis and the differentially expressed mRNAs analysis. Several tools were used to predict the miRNAs targeted by the circRNAs and the miRNAs targeted mRNAs, which then overlapped with the cocaine-associated differentially expressed mRNAs to determine the functional roles of circRNAs. Functional analysis for the obtained mRNAs was performed *via* Gene Ontology (GO) in Metascape database. Integrated bioinformatics analysis was conducted to further characterize the circRNA–miRNA–mRNA regulatory network and identify the functions of distinct circRNAs. We found a total of 41 differentially expressed circRNAs, and 98 miRNAs were targeted by these circRNAs. The overlapped mRNAs targeted by the miRNAs and the differentially expressed mRNAs constructed a circRNA–miRNA–mRNA regulation network including 24 circRNAs, 43 miRNAs, and 82 mRNAs in the dlPFC of patients with cocaine use disorder. Functional analysis indicated the regulation network mainly participated in cell response-related, receptor signaling-related, protein modification-related and axonogenesis-related pathways, which might be involved with cocaine use disorder. Additionally, we determined four hub genes (*HSP90AA1*, *HSPA1B*, *YWHAG*, and *RAB8A*) from the protein–protein interaction network and constructed a circRNA–miRNA-hub gene subnetwork based on the four hub genes. In conclusion, our findings provide a deeper understanding of the circRNAs-related ceRNAs regulatory mechanisms in the pathogenesis of cocaine use disorder.

## Introduction

Cocaine addiction inflicts enormous health and economic costs to individuals, families, and society (Reid et al., 2012; United Nations Office on Drugs and Crime, 2020). Recently, significantly increased studies have focused on the field of neuroscience of cocaine use disorder, but its neurobiological mechanism is still unclear, and there is no effective clinical treatment for cocaine use disorder (Gawin and Ellinwood, 1989; Majewska, 1996a,b; Heal et al., 2014).

Epigenetic mechanisms can integrate both genetic and diverse environmental stimuli to exert potent and often long-lasting changes in gene expression (Jaenisch and Bird, 2003). Accumulating research has found epigenetic mechanism plays an important role in the drug addiction (Robison and Nestler, 2011; Nestler, 2014; Nestler and Lüscher, 2019). Non-coding RNAs, specifically long non-coding RNAs, circular RNAs (circRNAs), and small non-coding RNAs, are one type of common epigenetic regulators that play a vital role in many biological processes associated with diseases (Amin et al., 2019; Mehta et al., 2020).

Circular RNAs are vastly conserved non-coding RNAs formed by back-splicing and covalent fusion of RNA free ends into natural circles (Vicens and Westhof, 2014; Szabo and Salzman, 2016; Greene et al., 2017; Li et al., 2018). Because circRNAs lack poly(A) tails and cap structure, they are not affected by RNA exonuclease (Vicens and Westhof, 2014; Szabo and Salzman, 2016; Greene et al., 2017; Li et al., 2018). CircRNAs usually exert their functions as transcriptional and post-transcriptional regulators through various functional mechanisms, such as RNA binding protein (RBP) “sponges” (Du et al., 2016; Holdt et al., 2016), translated proteins (Legnini et al., 2017; Pamudurti et al., 2017), and RNA–RNA interaction (Li et al., 2015). At present, circRNAs function mainly by absorbing microRNAs (miRNAs) as competing endogenous RNAs (ceRNAs) regulatory network to regulate their target genes expression, which construct a functional circRNA–miRNA–mRNA regulation network (Hansen et al., 2013; Vicens and Westhof, 2014; Rybak-Wolf et al., 2015; Du et al., 2017; Greene et al., 2017; Li et al., 2018; Mehta et al., 2020). For example, knockdown of circHIPK2 expression significantly inhibited astrocyte activation induced by methamphetamine through the targeting of miR124 and SIGMAR1 (Huang et al., 2017). Another study reported that circTmeff-1 promotes incubation of context-induced morphine craving by sponging miR-541/miR-6934 in the nucleus accumbens (Yu et al., 2021).

Although several circRNAs have been identified as participating in cocaine addiction, the regulatory networks in patients with cocaine use disorder are still unknown. It is necessary to conduct the circRNA–miRNA–mRNA regulatory networks in patients with cocaine use disorder to help to advance our understanding of the molecular mechanism of cocaine use disorder. Dorsolateral prefrontal cortex (dlPFC), similar role to medial PFC in rodents (Seamans et al., 2008), is a crucial component brain region of inhibitory control (Gass and Chandler, 2013; Moeller et al., 2014), which undergoes significant changes after long-term cocaine use (Matochik et al., 2003; Moreno-Lopez et al., 2012) and is involved in compulsive drug-seeking behaviors, increasing drug intake and addiction severity (Chen B. T. et al., 2013; Conti and Nakamura-Palacios, 2014; Terraneo et al., 2016). In this study, we aimed to investigate the functional circRNA–miRNA–mRNA regulatory networks in the dlPFC of patients with cocaine use disorder. Lastly, we constructed a circRNA–miRNA–mRNA regulation network including 24 circRNAs, 43 miRNAs, and 82 mRNAs, which may reveal a novel molecular mechanism in pathogenesis of patients with cocaine use disorder.

## Materials and Methods

### Data Collection

The circRNAs expression data were obtained from GSE99349 in GEO database.^1^ The data were generated using RNA sequencing (RNA-seq) of human postmortem dlPFC neuronal nuclei for 19 patients with cocaine use disorder and 17 unaffected controls. All patients who met criteria for cocaine use disorder were identified sudden deaths due to the toxic effects of chronic cocaine abuse (Ribeiro et al., 2017). Unaffected controls, who were selected from homicides, accidental or natural deaths, were drug-free age-matched subjects. Post-mortem interval (PMI), RNA integrity number (RIN), age, and race are provided in the original paper and do not significantly differ between cases and controls (Ribeiro et al., 2017). In the original study, the authors analyzed the differentially expressed genes and non-coding linear RNAs, but did not analyze the circRNAs. We further analyzed the circRNAs using the data of GSE99349 and used the differentially expressed genes in the original study to overlap predicted genes.

In addition, we collected some differentially expressed mRNAs from PFC RNA-seq data of different cocaine addiction animal models (GSE124952 and GSE89572) (Li et al., 2017; Bhattacherjee et al., 2019).

### Identification of Differentially Expressed Circular RNAs

Cutadapt (Martin, 2011) was used to remove the reads that contained adaptor contamination, low-quality bases, and undetermined bases. Next, sequence quality was verified using FastQC (Andrews, 2010). Bowtie 2 was used to map reads to the human genome hg37 (Langmead and Salzberg, 2012). CIRI2 was initially used for *de novo* assembly of the mapped reads into circRNAs (Gao et al., 2018); subsequently, back-splicing reads were identified in unmapped reads using CIRI2. The total reads and the number of mapped reads per sample is shown in Supplementary Table 1. The differentially expressed of circRNAs were calculated using R package edgeR (Robinson et al., 2010). Only the comparisons with *P*-value < 0.05 and fold change ≥ 1.5 were regarded as differential expressed circRNAs.

### Target MicroRNAs and mRNAs Prediction and Regulatory Network Establishment

MicroRNAs targeted by circRNAs were predicted using miRDB (target score >80) (Liu and Wang, 2019; Chen and Wang, 2020). Putative miRNAs were listed based on competitive binding ability, the top five miRNAs for each circRNA were mainly considered as circRNA target (Lv et al., 2018) and selected for further targeted mRNA predictions using TargetScan (score <−0.4) (Agarwal et al., 2015), DIANA-microT (score >0.8) (Paraskevopoulou et al., 2013), Tarbase (Vergoulis et al., 2012), and miRDB (score >80) (Liu and Wang, 2019). TargetScan, DIANA-microT, and TarBase are based on DIANA-miRPath v.3 platform (Fromm et al., 2015; Vlachos and Hatzigeorgiou, 2017). Only the target mRNAs presented in at least 3 out of 4 databases were considered as target genes of the given miRNAs. The targeted mRNAs were then overlapped with the differentially expressed mRNA data of the dlPFC neurons of patients with cocaine use disorder (Ribeiro et al., 2017). Last, a circRNA–miRNA–mRNA regulatory network was constructed. Cytoscape (Shannon et al., 2003) (version 3.6.0) was used to delineate the cocaine-related gene regulatory network.

### Gene Set Enrichment Analysis for mRNAs in the Regulatory Network

To assess functional enrichment, Metascape Gene Ontology (GO) terms were used to perform gene set enrichment analysis for the mRNAs in the circRNA–miRNA–mRNA network (Zhou et al., 2019). The thresholds of enrichment analysis were set as GO terms with *P* < 0.01 and the count of genes involved in the GO terms ≥3.

### Establishment of Protein–Protein Interaction Network and Identification of Hub Genes

The protein–protein interaction (PPI) network of the mRNAs in the circRNA–miRNA–mRNA network was established using the STRING database (Szklarczyk et al., 2017; Doncheva et al., 2019), and then visualized using Cytoscape software (Shannon et al., 2003). Subsequently, cytoHubba app (Chin et al., 2014) of Cytoscape was used to determine the hub genes. According to the degree ranks of cytoHubba app, the nodes degree ≥5 were considered as hub genes. The structure pattern of several vital circRNAs associated with hub genes were drawn using the database CSCD (Feng et al., 2021), which can be used for predicting miRNA response element, RBP, and open reading frame to better explore the potentially functional mechanisms of the selected circRNA.

## Results

### Identification of Differentially Expressed Circular RNAs in Dorsolateral Prefrontal Cortex of Patients With Cocaine Use Disorder

**FIGURE 1:**
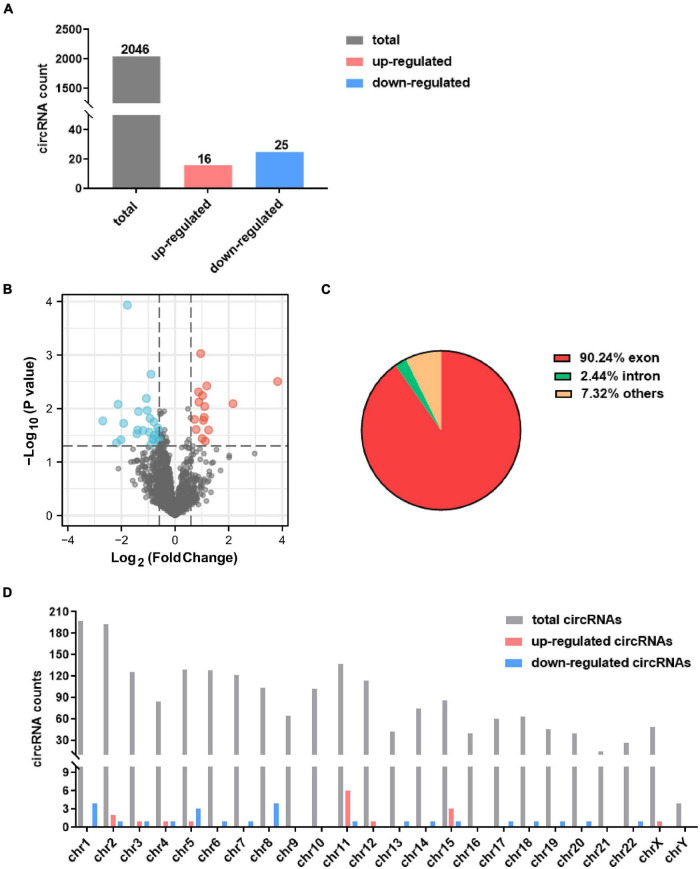
Identification of differentially expressed circRNAs in dorsolateral prefrontal cortex neurons of patients with cocaine use disorder. **(A)** The count of identified circRNAs. **(B)** Volcano plot showing circRNAs expression in patients with cocaine use disorder and unaffected controls. The red and blue dots represent circRNAs with statistically significant differences in expression. **(C)** Pie chart showing the percentage of circRNAs derived from different genomic regions. **(D)** Distributions of identified circRNAs along the chromosomes.

A total 2,046 circRNAs were identified in the GSE99349 dataset, and exon-derived circRNA account for 77.4% (Figure 1A). Among these, 16 up-regulated circRNAs and 25 down-regulated circRNAs with fold changes ≥ 1.5 and *P*-values ≤ 0.05 were considered as significantly differentially expressed circRNAs (Figure 1B). Among the differentially expressed circRNAs, 65.9% had already existed in the circBase database (Glažar et al., 2014), 14 were *de novo* significantly differentially expressed circRNAs (Tables 1, 2). Of the differentially expressed circRNAs, 90.24% were covered in the exon of the genome (Figure 1C), others aligned with intron or other sequences. Interestingly, non-coding RNA MALAT1 produced seven circRNAs (named circMALAT1-1 to circMALAT1-7 in Tables 1, 2). Additionally, the chromosome distribution of the circRNAs showed no significant differences (Figure 1D).

**TABLE 1:** Basic characteristics of the up-regulated circRNAs.

CircRNA_name	circBase_ID	Ensemble_ID	Log fold_change	*P*_value	Genome_location
circPAPSS1	hsa_circ_0005965	ENSG00000138801	3.826226	0.00312	chr4:108603171| 108615162
circEIF3J	–	ENSG00000104131	2.162914	0.008119	chr15:44843074| 44846865
circDBN1	–	ENSG00000113758	1.249996	0.025277	chr5:176887645| 176893833
circSLC30A6	hsa_circ_0005695	ENSG00000152683	1.126922	0.040697	chr2:32399132| 32409407
circERC2	hsa_circ_0124267	ENSG00000187672	1.105095	0.00919	chr3:55984453| 56026278
circSCAPER	hsa_circ_0000640	ENSG00000140386	1.088051	0.014667	chr15:77020936| 77025725
circGRIN2B	–	ENSG00000273079	1.024252	0.005729	chr12:13708789| 13708961
circMALAT1-1	–	ENSG00000251562	0.955983	0.000941	chr11:65267096| 65267394
circMALAT1-2	–	ENSG00000251562	1.182962	0.003779	chr11:65267060| 65267236
circMALAT1-3	–	ENSG00000251562	0.722058	0.015946	chr11:65267954| 65268132
circMALAT1-4	–	ENSG00000251562	1.056787	0.016663	chr11:65267237| 65267385
circMALAT1-5	–	ENSG00000251562	0.786989	0.024681	chr11:65266605| 65266756
circMALAT1-6	–	ENSG00000251562	1.014213	0.036447	chr11:65266720| 65266894
circSRBD1	hsa_circ_0120146	ENSG00000068784	0.89058	0.007569	chr2:45773871| 45812913
circMYO5A	hsa_circ_0103878	ENSG00000197535	0.877909	0.004901	chr15:52638558| 52646211
circIL1RAPL1	–	ENSG00000169306	0.666318	0.050085	chrX:28941541| 28943776

*The symbol “–” indicating this circRNA was not existing in circBase database.*

**TABLE 2:** Basic characteristics of the down-regulated circRNAs.

CircRNA_name	circBase_ID	Ensemble_ID	Log fold_change	*P*_value	Genome_location
circMALAT1-7	–	ENSG00000251562	−0.59212	0.038581	chr11:65267160| 65267534
circEGLN1	hsa_circ_0000196	ENSG00000135766	−0.59753	0.026098	chr1:231506308| 231509845
circSATB1	hsa_circ_0064557	ENSG00000182568	−0.61692	0.041324	chr3:18456603| 18462483
circKHDRBS3	hsa_circ_0135838	ENSG00000131773	−0.61726	0.030379	chr8:136533480| 136569830
circLRCH1	hsa_circ_0002215	ENSG00000136141	−0.64777	0.023012	chr13:47297356| 47308133
circRGS7-1	hsa_circ_0112723	ENSG00000182901	−0.77351	0.031668	chr1:240990398| 241033419
circSATB2	hsa_circ_0003915	ENSG00000119042	−0.79387	0.017806	chr2:200233328| 200298237
circMNAT1	hsa_circ_0008215	ENSG00000020426	−0.79855	0.038421	chr14:61278705| 61346553
circRBM39	hsa_circ_0005848	ENSG00000131051	−0.83443	0.036584	chr20:34309662| 34320057
circHOOK3	hsa_circ_0005376	ENSG00000168172	−0.89529	0.002289	chr8:42780700| 42798588
circAKAP10	hsa_circ_0006256	ENSG00000108599	−0.9144	0.047896	chr17:19812494| 19813291
circSNTG1	–	ENSG00000147481	−0.93671	0.015294	chr8:51362228| 51503477
circATXN10	hsa_circ_0003054	ENSG00000130638	−0.96313	0.027635	chr22:46085592| 46114373
circESCO1	hsa_circ_0047071	ENSG00000141446	−1.03841	0.010851	chr18:19112434| 19112621
circSTXBP5-AS1	–	ENSG00000233452	−1.3646	0.011378	chr6:147394380| 147395983
circTJP1	hsa_circ_0034293	ENSG00000104067	−1.06956	0.006483	chr15:30053342| 30065560
circCAP1	hsa_circ_0009142	ENSG00000131236	−1.19278	0.0257077	chr1:40529899| 40530231
circMTHFD2L	hsa_circ_0069982	ENSG00000163738	−1.39497	0.025271	chr4:75040223| 75091111
circRGS7-2	hsa_circ_0007091	ENSG00000182901	−1.41979	0.029781	chr1:241094017| 241100006
circADAMTS19	hsa_circ_0073810	ENSG00000145808	−1.78036	0.000117	chr5:128861977| 128887600
circARHGAP26	hsa_circ_0074368	ENSG00000145819	−1.915	0.018909	chr5:142416761| 142437312
circRASA1	hsa_circ_0004317	ENSG00000145715	−2.01716	0.038434	chr5:86627165| 86649052
circLUC7L2	hsa_circ_0133534	ENSG00000146963	−2.12507	0.008356	chr7:139083345| 139097326
circCSPP1	hsa_circ_0084665	ENSG00000104218	−2.18439	0.044077	chr8:68007528| 68007967
*	–	Intergenic	−2.69379	0.017096	chr19:11977352| 12058122

The symbol “*” indicating this circRNA was from intergenic region. The symbol “–” indicating this circRNA was not existing circBase database.

### Construction of the circRNA–miRNA–mRNA ceRNAs Network

**FIGURE 2:**
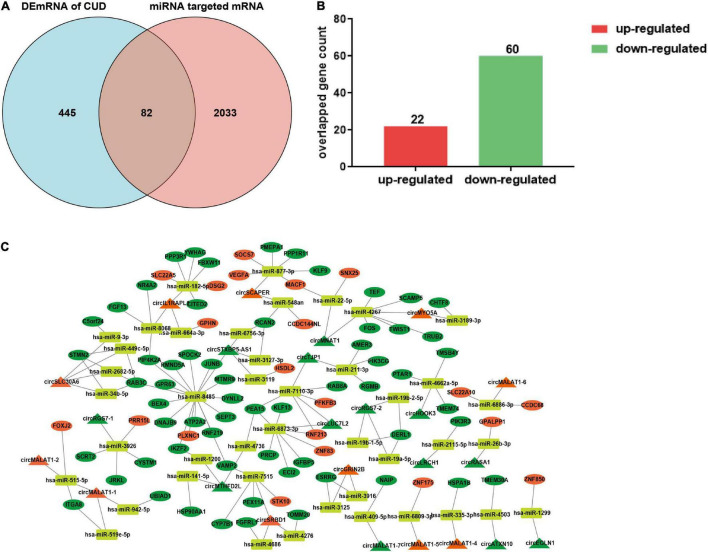
Construction of the ceRNA network. **(A)** The shared genes between differentially expressed mRNAs in dlPFC neurons of patients with cocaine use disorder and predicted mRNAs using the miRNAs targeted by circRNAs. **(B)** Histogram showing the up-regulated mRNAs and down-regulated mRNAs in shared genes. **(C)** The circRNA–miRNA–mRNA regulatory network. The triangle, ellipse, rectangle, respectively presents circRNAs, mRNAs, and miRNAs. Orange triangle and ellipse represents up-regulated circRNAs and mRNAs, respectively; green triangle and ellipse represents down-regulated circRNAs and mRNAs, respectively. DEmRNAs, differentially expressed mRNAs; CUD, cocaine use disorder.

Given the potential regulatory roles of circRNAs on recruiting miRNAs to regulate the expression of target genes, we predicted the miRNA “sponges” of circRNA using miRDB database, and 98 miRNAs were found to be closely targeted by the differentially expressed circRNAs. These 98 miRNAs further targeted 2,115 mRNA genes, among which, 82 mRNAs were overlapped with the differentially expressed mRNA of dlPFC neurons of patients with cocaine use disorder (Ribeiro et al., 2017), including 22 up-regulated mRNAs and 60 down-regulated mRNAs (Figures 2A,B). Ultimately, the 82 target mRNAs were targeted by 43 miRNAs, and the miRNAs were further targeted by 24 circRNAs, which formed a circRNA–miRNA–mRNA network for further study (Figure 2C).

### Functional and Pathway Enrichment Analyses

**FIGURE 3:**
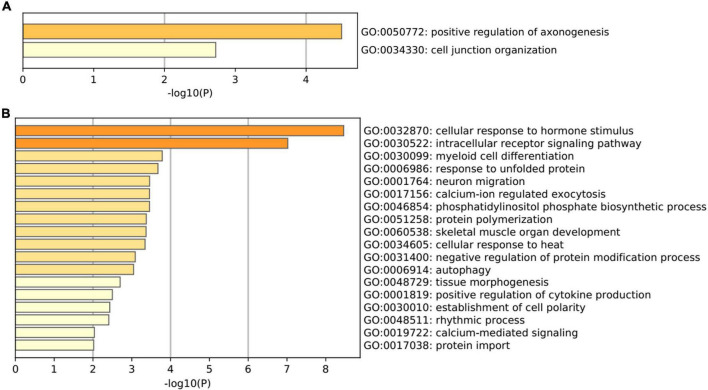
Gene Ontology terms enriched by the shared genes between differentially expressed mRNAs in dlPFC neurons of patients with cocaine use disorder and predicted mRNAs using the miRNAs targeted by circRNAs. **(A)** GO terms enriched by the up-regulated mRNAs. **(B)** GO terms enriched by the down-regulated mRNAs.

Gene Ontology pathway enrichment analysis for the 82 genes aberrantly expressed in the patients with cocaine use disorder and indirectly regulated by circRNAs revealed that the 22 up-regulated genes participated in vital biological processes including positive regulation of axonogenesis (GO: 0050772) and cell junction organization (GO: 0034330) (Figure 3A), which is consistent with current reports on the relationship between cocaine addiction and the synaptic transmission (Li et al., 2021; Wang et al., 2021; Zinsmaier et al., 2021). Moreover, the 62 down-regulated genes participated in vital biological processes including cellular response (GO:0032870, GO:0034605, and GO:0048511), protein modification and transport process (GO:0006986, GO:0046854, GO:0051258, GO:0031400, and GO:0017038), intracellular receptor and calcium-ion (GO:0030522, GO:0017156, and GO:0019722), cell and tissue morphogenesis (GO:0030099, GO:0001764, GO:0060538, GO:0048729, and GO:0030010), autophagy (GO:0006914), and positive regulation of cytokine production (GO:0001819) (Figure 3B), which implied that the etiology of cocaine use disorder may involve many biological processes.

### Establishment of Protein–Protein Interaction Network and Identification of Hub Genes

**FIGURE 4:**
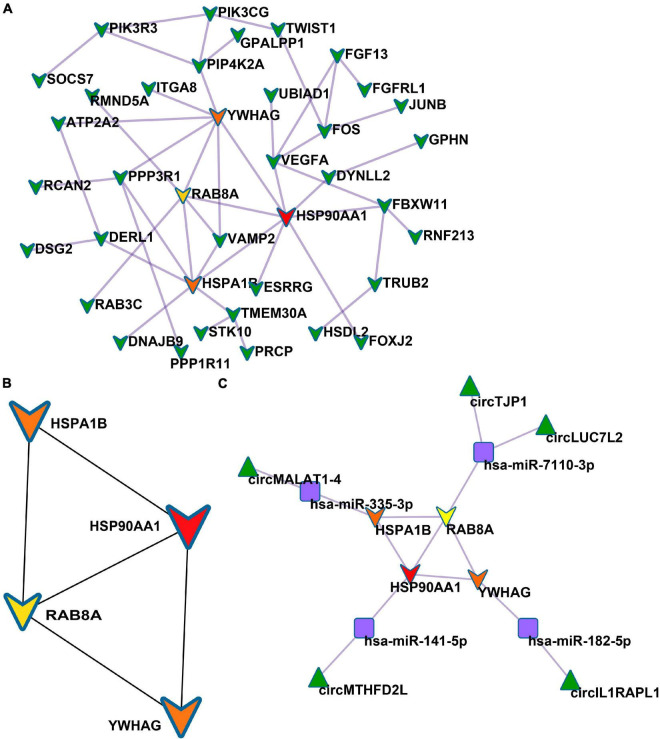
Identification of hub genes from the PPI network. **(A)** PPI network containing 38 nodes and 47 edges. **(B)** PPI network of four hub genes that extracted from the PPI network. **(C)** The circRNA–miRNA-hub gene network.

**FIGURE 5:**
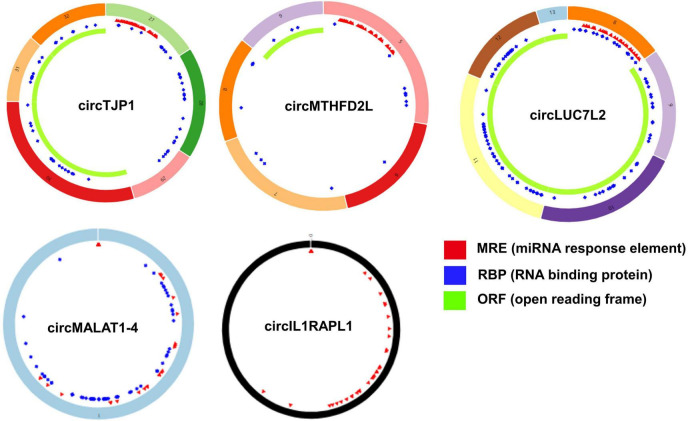
Structural patterns of the five important circRNAs from circRNA–miRNA-hub gene network. Structural patterns of circTJP1, circMTHFD2L, circLUC7L2, circMALAT1-4, and circIL1RAPL1. The colored circle represents the circRNAs that consist of exons. The numbers on the circRNAs mean the exon number. The red, blue, and green regions inside the circRNA molecule, respectively represent MRE (microRNA response element), RBP (RNA binding protein), and ORF (open reading frame).

Based on the STRING database, among the 82 genes aberrantly expressed in the patients with cocaine use disorder and indirectly regulated by circRNAs, 38 genes formed a PPI network, containing 38 nodes and 47 edges (Figure 4A). The highest-scoring nodes (degree ≥5) were screened as hub genes: *HSP90AA1*, *HSPA1B*, *YWHAG*, and *RAB8A* (Figure 4B). It is well known that hub nodes with high degrees of connectivity have vital functions in biological networks (Han et al., 2004; Wang et al., 2018). Hence, we used these genes to construct a circRNA–miRNA-hub gene subnetwork (Figure 4C): circMTHFD2L/hsa-miR-141-5p/*HSP90AA1*, circMALAT1-4/hsa-miR-335-3p/*HSPA1B*, circIL1RAPL1/hsa-miR-182-5p/*YWHAG*, circTJP1/hsa-miR-7110-3p/*RAB8A*, and circLUC7L2/hsa-miR-7110-3p/*RAB8A*. Based on the circRNA–miRNA-hub gene subnetwork, five circRNAs (circMTHFD2L, circMALAT1-4, circIL1RAPL1, circTJP1, and circLUC7L2) were likely to play important roles in cocaine use disorder. In order to further recover the function of the five vital circRNAs, the structural patterns of these vital circRNAs were shown in Figure 5.

### Circular RNA–MicroRNA–mRNA Network Regulation in Different Cocaine Addiction Animal Model

**FIGURE 6:**
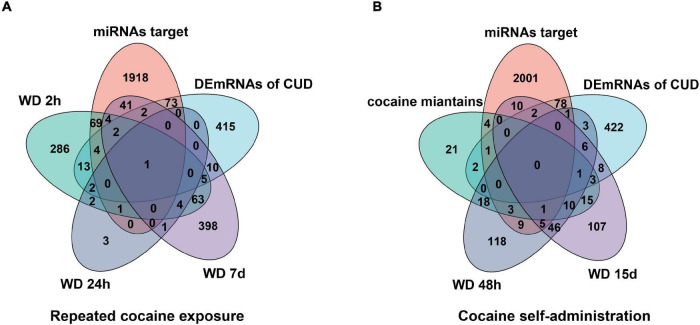
Overlap analysis for the mRNAs from the circRNAs–miRNA–mRNA network with the differentially expressed genes in different cocaine addiction animal model. **(A)** The shared genes between mRNAs in ceRNA network and differentially expressed mRNAs in different withdrawal time points after repeated cocaine exposure. **(B)** The shared genes between mRNAs in ceRNA network and differentially expressed mRNAs in different withdrawal time points after cocaine self-administration. miRNA target, predicted mRNAs using miRNAs targeted by circRNAs; human mRNA, differentially expressed mRNAs in dlPFC neuron of human with cocaine use disorder; WD 2h, withdrawal 2 h; WD 24h, withdrawal 24h; WD 48 h, withdrawal 48h; WD 7d, withdrawal 7 days; WD 15d, withdrawal 15 days.

To further verify the “sponge” function of circRNAs in cocaine addiction, we utilized the differentially expressed mRNAs from PFC RNA-seq data of different cocaine addiction models to overlap with the predicted mRNA indirectly regulated by circRNAs and differentially expressed mRNA in dlPFC of patients with cocaine use disorder. Many genes participate in the circRNA–miRNA–mRNA network regulation (Figure 6A) in the chronic cocaine exposure models with different withdrawal time points. However, only one gene, *FOS*, was overlapped in all different withdrawal time points (Table 3). Similarly, there were many genes in the circRNAs–miRNA–mRNA network involving in cocaine self-administration model (Figure 6B). As shown in Table 4, hub gene *YWHAG* and *HSP90AA1* participated in cocaine maintains and withdrawal 15 days, respectively.

**TABLE 3:** The shared genes between mRNAs in ceRNA network and differentially expressed mRNAs in different withdrawal time points after repeated cocaine exposure.

mRNAs in ceRNA network overlapped with WD 2 h	mRNAs in ceRNA network overlapped with WD 24 h	mRNAs in ceRNA network overlapped with WD 7 days
*PFKFB3*	*FOS*	*SPOCK2*
*KLF9*		*STMN2*
*KLF13*		*KLF13*
*SOCS7*		*SOCS7*
*NR4A2*		*FOS*
*JUNB*		
*FOS*		

*WD, withdrawal.*

**TABLE 4:** The shared genes between mRNAs in ceRNA network and differentially expressed mRNAs in different withdrawal time points after cocaine self-administration.

mRNAs in ceRNA network overlapped with cocaine maintains	mRNAs in ceRNA network overlapped with WD 48 h	mRNAs in ceRNA network overlapped with WD 15 days
*YWHAG*	*STMN2*	*TMEM30A*
		*HSP90AA1*

*WD, withdrawal.*

## Discussion

Most of previous research on the mechanism of addiction was based on animal models, or the peripheral blood of patients with substance use disorders; however, the studies do not truly portray the changes that occur in the brains of patients with substance use disorders, which may be an important obstruction to the study of drugs for the treatment of substance use disorders. The circRNA–miRNA–mRNA regulatory network we constructed will enhance the understanding of the addiction mechanism in the brain of patients with cocaine use disorder.

Through the functional enrichment analysis of the mRNAs in the network, we found that the up-regulated mRNAs were mainly involved in regulation of axonogenesis and cell junction, which suggests that our up-regulated mRNA may have a close connection with synaptic transmission, and previous studies have confirmed that abnormal synaptic transmission is a very critical factor for cocaine addiction (Khibnik et al., 2016; Martínez-Rivera et al., 2017; Li et al., 2021). Down-regulated mRNAs were found to be involved in many biological processes, including cellular response to hormone stimulus, response to unfolded protein, cellular response to heat, intracellular receptor signaling pathway, myeloid cell differentiation, calcium-ion regulated exocytosis, calcium-mediated signaling, and autophagy. All the biological processes related to down-regulated mRNAs in the circRNA–miRNA–mRNA regulatory network have been involved with cocaine addiction. For example, clinical trials have investigated that cocaine associated cues could significantly increase adrenocorticotropic hormone and cortisol (Berger et al., 1996). A single dose of cocaine can cause the accumulation of different heat shock proteins (Salminen et al., 1997), which leads to blood-brain barrier breakdown and brain edema formation thereby promoting cocaine intoxication (Sharma et al., 2009). Apart from this, Cocaine has the propensity to cause hyperthermia which increases the mortality rates to cocaine (Crandall et al., 2002). These are also evidences reported that various intracellular receptors, especially dopamine receptors and glutamate receptors, are all critical for cocaine addiction (Ellenbroek, 2013; Howell and Cunningham, 2015; Smaga et al., 2019). In addition, brain myeloid cells, particularly microglia, presented in the brain parenchyma, serve as a surveillance function for neuroinflammation and neurodegeneration in the central nervous system (Ransohoff and Cardona, 2010; Ajami et al., 2018; Jordão et al., 2019). Addictive drugs, especially cocaine, have been consistently shown to activate microglia both *in vitro* and *in vivo* (Guo et al., 2015; Liao et al., 2016). In rodents, inhibiting glial cell activation was shown to block cocaine-mediated behavioral changes (Chen et al., 2009). In humans, cocaine exposure can reduce microglial cells viability and inhibit the expression of extracellular vesicle-associated proteins disrupting cellular signaling and cell-to-cell communication (Kumar et al., 2020). Therefore, it can be considered that circRNAs were thought to play an important role in the multiple addiction-related networks in dlPFC of patients with cocaine use disorder.

Circular RNAs are endogenous non-coding RNAs with widespread distribution and various cellular function (Hansen et al., 2013; Vicens and Westhof, 2014; Rybak-Wolf et al., 2015; Du et al., 2017; Greene et al., 2017; Li et al., 2018; Mehta et al., 2020). Numerous studies have shown that circRNAs have an important influence on many complicated neuropsychiatric disorders (Cui et al., 2016; Zhang et al., 2018; An et al., 2019; Liu et al., 2019; Mahmoudi et al., 2019; Huang et al., 2020; Zhang Y. et al., 2020; Zimmerman et al., 2020), including drug addiction (Huang et al., 2017; Bu et al., 2019; Li et al., 2019, 2020; Zhang H. et al., 2020). Knockdown of circHomer1 ameliorates methamphetamine-induced neuronal injury through inhibiting Bbc3 expression (Li et al., 2020). The abnormal expression of mmu_circRNA_002381 in striatum was induced by cocaine self-administration and cocaine-induced locomotor activity model (Bu et al., 2019). Interestingly, siRNA-mediated mmu_circRNA_002381 down-regulation increased the expressions of *limk1* and *bdnf*, which are the targets of miR-138 associated with synaptic plasticity. Additionally, some studies predicted that circRNAs are involved in the progress and development of many addictive drug models by sponging miRNA to regulate downstream targets (Li et al., 2017, 2020; Bu et al., 2019; Zhang H. et al., 2020). In our study, 24 circRNAs were identified to be involved in the circRNA–miRNA–mRNA regulatory network. Among these, 15 were identified previously in the mammalian brain as being dysregulated during neuronal differentiation and highly enriched in synapses (Rybak-Wolf et al., 2015). It was suggested that circRNAs in the circRNA–miRNA–mRNA regulatory network may play their regulatory functions in the neurons or synapses of patients with cocaine use disorder.

Several studies have revealed that circRNAs contain multiple miRNA response elements and can bind to miRNAs, often termed as “miRNA sponges,” decreasing cytoplasmic levels of miRNAs and liberating their respective downstream target mRNAs (Memczak et al., 2013; Piwecka et al., 2017; Kleaveland et al., 2018; Zhang Y. et al., 2020). Here, we constructed a circRNA–miRNA–mRNA regulation network involved in cocaine use disorder, including 24 circRNAs, 42 miRNAs, and 82 mRNAs. CircSLC30A6 was down-regulated in the dlPFC of patients with cocaine use disorder. Based on the analysis of circRNA–miRNA–mRNA network, we found that circSLC30A6 interacts with hsa-miR-9-3p. Interestingly, it has been known that hsa-miR-9-3p mediates the dynamic regulation of neural progenitor proliferation during neurogenesis (Pascale et al., 2020). Moreover, hsa-miR-9-3p is significantly increased in serums of patients with methamphetamine use disorder compared with normal controls (Gu et al., 2020). Therefore, we assume that circSLC30A6 up-regulation induced by the cocaine may be involved in cocaine effect through interacting with hsa-miR-9-3p. CircRASA1 and circMNAT1 were down-regulated in the dlPFC of patients with cocaine use disorder. Through the analysis of circRNA–miRNA–mRNA network, we found that circRASA1 interacts with hsa-miR-26b-3p and circMNAT1 interacts with hsa-miR-22-5p. miR-26b was found to be up-regulated in hippocampus following the acquisition and extinction but miR-22 was only up-regulated during extinction of cocaine-induced conditioned place preference in rats (Chen C. L. et al., 2013). Therefore, it is conceivable that circSLC30A6, circRASA1, and circMNAT1 might play an important role in cocaine use disorder through modulating their target miRNA.

To further identify the key circRNAs participating in the regulatory network, we established a PPI network and screened four hub genes, including *HSP90AA1*, *HSPA1B*, *YWHAG*, and *RAB8A*. Accordingly, we constructed a circRNA–miRNA-hub gene subnetwork. In the cocaine self-administration model, *HSP90AA1* had a significant decrease in PFC after withdrawal of 15 days (Bhattacherjee et al., 2019), however, the expression of *HSP90AA1* in posterior hippocampus increased significantly after 28 days of withdrawal (García-Fuster et al., 2012). These delayed neurobiological effects of *HSP90AA1* likely contribute to sustained vulnerability to cocaine relapse, which may be regulated by circMTHFD2L. *HSPA1B* gene is one of heat shock protein 70 (HSP70)-encoding transcripts, and it is reported that *HSPA1B* expression was increased in the postmortem brains of patients with cocaine use disorder exhibiting excited delirium in comparison with other (non-excited delirium) cocaine-related deaths and drug-free controls, concluding that elevated *HSPA1B* provides a reliable forensic biomarker for the identification of excited delirium (Mash et al., 2009; Johnson et al., 2012). The structure of circRNA makes them more stable and has a longer half-life, so it is considered to be a more ideal marker (Enuka et al., 2016). Previous studies reported that cocaine exposure dysregulated the expression of *YWHAG* (Bhattacherjee et al., 2019), and reduced *YWHAG* can lead to neuronal hyperexcitability, and normalization of hyperexcitability can rescue memory deficits (Roy et al., 2021). RAB8A is a member of the RAS superfamily, which are key regulators of intracellular membrane trafficking from the formation of transport vesicles to their fusion with membranes, and involve in polarized vesicular trafficking, and neurotransmitter release (Núñez et al., 2009; Esseltine et al., 2012; Sellier et al., 2016; Nüchel et al., 2018). Although no studies have shown that RAB8A has a direct effect on cocaine addiction, the biological process in which it participates is very important in cocaine addiction (Periyasamy et al., 2016; Harraz et al., 2021). Here, we identified five circRNA–miRNA-hub gene axes, indicating competitive regulatory relationships of five circRNAs with the four genes in cocaine use disorder. Nevertheless, the expression of downstream genes may be regulated by multiple circRNAs and miRNAs, the expression of the five key circRNAs may be not significantly correlated with its potential downstream gene targets in the sequencing data. Cocaine use disorder is a complex brain disease in which many factors, such as cell subtype specificity, synaptic plasticity, and neural circuit, can influence genes expression. The bioinformatics analysis, which integrated several datasets, could only provide a possible research direction, how the circRNAs contributes to the specific mechanism of cocaine use disorder requires more in-depth studies.

## Conclusion

In conclusion, our research is the first to use dlPFC circRNAs and mRNA of patients with cocaine use disorder *via* bioinformatic tools to identify a circRNA–miRNA–mRNA regulatory network in the patients with cocaine use disorder. The circRNA–miRNA-hub genes regulatory sub-network uncovered five important circRNAs that might be involved in cocaine use disorder, providing new insight into the pathogenesis of cocaine use disorder and suggesting potential therapeutic targets that warrant further investigation.

## Data Availability Statement

Publicly available datasets were analyzed in this study. This data can be found here: http://www.ncbi.nlm.nih.gov/geo/, GSE99349, GSE124952, and GSE89572.

## Author Contributions

YC, JS, and SC contributed toward conception and design of research. YC and XL analyzed the data and wrote the manuscript together. YC, JS, SM, SC, XL, and SH interpreted the results and revised the manuscript. All authors have read and approved the final version of the manuscript.

## Conflict of Interest

The authors declare that the research was conducted in the absence of any commercial or financial relationships that could be construed as a potential conflict of interest.

## Publisher’s Note

All claims expressed in this article are solely those of the authors and do not necessarily represent those of their affiliated organizations, or those of the publisher, the editors and the reviewers. Any product that may be evaluated in this article, or claim that may be made by its manufacturer, is not guaranteed or endorsed by the publisher.

## Footnotes

1
http://www.ncbi.nlm.nih.gov/geo/
