# Supplementary material for: Identification of Functional CircRNA–miRNA–mRNA Regulatory Network in Dorsolateral Prefrontal Cortex Neurons of Patients With Cocaine Use Disorder
Source: Front Mol Neurosci. 2022 Apr 14;15:839233. doi: 10.3389/fnmol.2022.839233 (PMC9048414; doi:10.3389/fnmol.2022.839233)
Supplement: Supplementary file 1 [file Table_1.DOCX]

|  | **Unaffected control** | **Patients with cocaine use disorder** |
| --- | --- | --- |
| the reads on circRNAs per sample | 2,307,775±264,877 (2.16%) | 1,931,745±334,757 (1.93%) |
| the total reads per sample | 106,415,431±15,930,326 | 100,325,045±10,064,111 |

Table S1. The total reads and the reads of mapping on circRNAs per sample
